# Simultaneous lipid production for biodiesel feedstock and decontamination of sago processing wastewater using *Candida tropicalis* ASY2

**DOI:** 10.1186/s13068-020-01676-1

**Published:** 2020-03-05

**Authors:** Kiruthika Thangavelu, Pugalendhi Sundararaju, Naganandhini Srinivasan, Iniyakumar Muniraj, Sivakumar Uthandi

**Affiliations:** 1 Department of Renewable Energy Engineering, Agricultural Engineering College and Research Institute, Coimbatore, Tamil Nadu 641 003 India; 2 Biocatalysts Lab, Department of Agricultural Microbiology, Tamil Nadu Agricultural University, Coimbatore, Tamil Nadu 641 003 India; 3 Department of Crop Management, Kumaraguru Institute of Agriculture, Erode, Tamil Nadu 641003 India

**Keywords:** Sago wastewater, Starch, Oleaginous yeast, Lipid production, Biodiesel

## Abstract

**Background:**

Without sufficient alternatives to crude oil, as demand continues to rise, the global economy will undergo a drastic decline as oil prices explode. Dependence on crude oil and growing environmental impairment must eventually be overcome by creating a sustainable and profitable alternative based on renewable and accessible feedstock. One of the promising solutions for the current and near-future is the substitution of fossil fuels with sustainable liquid feedstock for biofuel production. Among the different renewable liquid feedstock’s studied, wastewater is the least explored one for biodiesel production. Sago wastewater is the byproduct of the cassava processing industry and has starch content ranging from 4 to 7%. The present investigation was aimed to produce microbial lipids from oleaginous yeast, *Candida tropicalis* ASY2 for use as biodiesel feedstock and simultaneously decontaminate the sago processing wastewater for reuse. Initial screening of oleaginous yeast to find an efficient amylolytic with maximum lipid productivity resulted in a potent oleaginous yeast strain, *C. tropicalis* ASY2, that utilizes SWW as a substrate. Shake flask experiments are conducted over a fermentation time of 240 h to determine a suitable fatty acid composition using GC-FID for biodiesel production with simultaneous removal of SWW pollutants using ASY2.

**Results:**

The maximum biomass of 0.021 g L^−1^ h^−1^ and lipid productivity of 0.010 g L^−1^ h^−1^ was recorded in SWW with lipid content of 49%. The yeast strain degraded cyanide in SWW (79%) and also removed chemical oxygen demand (COD), biological oxygen demand (BOD), nitrate (NO_3_), ammoniacal (NH_4_), and phosphate (PO_4_) ions (84%, 92%, 100%, 98%, and 85%, respectively). GC-FID analysis of fatty acid methyl esters (FAME) revealed high oleic acid content (41.33%), which is one of the primary fatty acids for biodiesel production.

**Conclusions:**

It is evident that the present study provides an innovative and ecologically sustainable technology that generates valuable fuel, biodiesel using SWW as a substrate and decontaminates for reuse.

## Background

Interest in renewable biofuels as a substitute for petroleum-derived fuels has risen progressively over the last several decades. The global biodiesel market is estimated to reach 37 billion gallons by 2016, with an annual growth rate of 42% [1]. Biodiesel, a green fuel, can be produced from numerous feedstocks, including soil-based crops, microbes, and waste grease comprising free fatty acids or triacylglycerides [2]. More than 95% of global biodiesel is made from vegetable oils like soybean, canola, corn, and rapeseed oils. Soybean oil is the most important biodiesel feedstock with 659 million pounds consumption in May 2019 followed by corn oil (144 million pounds) and canola oil [3]. According to the global industrial scenario, biodiesel is predominantly produced from oilseed plants (e.g., soybeans), which are less used in food products due to several limitations such as oxidative stability and low oil yield [4]. In order to overcome the issues faced in the first and second-generation biofuel, a suitable feedstock for biofuel production that must not be competitive with food products have to be explored. Besides, the feedstock should be readily available and sustainable [5].

Single-cell oil (SCO) from oleaginous microbes has been investigated as an alternative to vegetable oils for biodiesel production. Oils from oleaginous microorganisms, including microalgae, yeast, bacteria, and mold [6], are now regarded as promising candidates owing to the features such as being non-affected by seasonal differences, elevated lipid contents, capacity to be produced from a broad range of carbon sources, and similarity with vegetable oils in fatty acid composition [7]. Among the different sources, only microalgae have been extensively investigated to date. Although algae are theoretically considered excellent, significant technical hurdles such as shortage of appropriate land area for cultivation, low productivity levels, lack of optimal photosynthetically active radiation, prohibitive expenses of complementary light, temperature control, and protection against aggressive non-lipid producing species, and difficulties in separation prevent their cost-effective utilization [8].

Oleaginous yeasts offer an incredible alternative to microalgae for biodiesel production owing to the ability to easily scale up without seasonal and climatic variations, short life cycle, and high cell densities on a variety of low-cost raw materials, such as industrial wastewater containing sugars [9], agricultural wastes [10], raw glycerol [11] and wastewater streams [12]. Among these, wastewater remains the least explored source of microbial lipids.

Sago wastewater (SWW) is an effluent discharged from cassava-based starch industries during sago processing in South India, especially in Tamil Nadu. There are about 800 small-scale sago processing plants that discharge 40,000 to 50,000 L of SWW and 15 to 30 tons of sludge per day [13]. Sago processing industries produce two types of wastewater. The first type is released by the washing and peeling of cassava tubers and it has low chemical oxygen demand (COD). The second type is released during the extraction of starch, which has a high pollution load due to high COD and biological or biochemical oxygen demand (BOD) and contains starch up to 7% [14]. Owing to its starchy nature, SWW serves as an outstanding source for the production of microbial lipids by the oleaginous microorganisms. However, a high level of COD and other contaminants, including cyanide, require its treatment before reuse, which also curtails down the pollution load. To date, many technologies have been developed to treat SWW. Physical and chemical methods are not much promising due to the problem of residual sludge disposal. Biological methods require aeration and anaerobic processes at high rates such as anaerobic filter, hybrid upflow anaerobic sludge blanket (UASB) reactor, anaerobic rotating biological contactor, and fluidized bed systems for generation of biogas, electricity, and biohydrogen [15, 16]. Though biohydrogen has the highest calorific value (150 kJ g^−1^), its use as a domestic fuel is limited due to high combustibility [17]. Similarly, biomethane (calorific value of 45 kJ g^−1^) contributes to a global warming potential of 28–36 GWP over 100 years, which limits the use of SWW as a substrate [18]. Even though biodiesel has low calorific value (32.81 kJ g^−1^), it has many advantages over biohydrogen and biomethane such as less processing time, zero net emission, valuable co-products, and lipid production along with the treatment system.

Considering the associated pollution problems, availability, and reusability, the aim of the present study was to evaluate SWW for intracellular neutral lipid (predominantly triglycerides; TAG) accumulation in the oleaginous yeast *C. tropicalis* ASY2 for biodiesel production and to characterize FAME (fatty acid methyl ester) using FTIR (Fourier transform-infrared) and GC-FID (gas chromatography-flame ionization detector). This paper reveals the possibility of using cheaper, significant, and untapped industrial wastewater as a potential resource for the production of biodiesel besides the sequestration of the potential pollutants.

## Results and discussion

### Physicochemical characteristics of SWW

The physicochemical analysis of SWW revealed that the characteristics of wastewater mainly depend on processing steps and raw materials (Table 1). The color of SWW was either dull white or dirty white due to the presence of residual starch, and it had an unpleasant odor. The pH was acidic (4.67) due to the presence of hydrogen cyanide (HCN) released from cassava tubers and sulfuric acid added during the extraction process. The electrical conductivity (EC) of SWW was 6.30 dS m^−1^. Nutrients, such as nitrogen (ammoniacal and nitrate) and phosphorus, were sufficient to support microbial growth. We recorded 3.10 mg L^−1^, 5.48 mg L^−1^, and 611.67 mg L^−1^ of ammoniacal nitrogen, nitrate-nitrogen, and phosphate levels, respectively. The total nitrogen content observed was 0.54 g L^−1^. The total solids (4.57 g L^−1^) and total dissolved solids (4.16 g L^−1^) of SWW were high due to the presence of colloidal starch particles, insoluble starch, scraps, and fibers [19]. The BOD and COD of SWW were 5.04 g L^−1^ and 70.67 g L^−1^, respectively, which indicate the high organic nature of wastewater. The starch content of SWW varied with the type of industry.

**Table 1:** Physicochemical characteristics of raw SWW

SWW parameters	Values (g L^−1^)
pH	4.67 (± 0.03)
EC dS m^-1^	6.30 (± 0.04)
Salinity	4.86 (± 0.01)
TDS	4.16 (± 0.02)
TS	4.57 (± 0.01)
Starch	10.00 (± 0.07)
TN	0.54 (± 0.001)
BOD	5.04 (± 0.08)
COD	70.67 (± 0.06)
NO_3_ (mg L^−1^)	3.10 (± 0.02)
NH_4_ (mg L^−1^)	5.48 (± 0.05)
PO_4_ (mg L^−1^)	611.67 (± 0.01)
Cyanide (mg L^−1^)	4.46 (± 0.02)

*EC* electrical conductivity, *dS m*^*-1*^deciSiemens per meter, *TDS* total dissolved solids, *TS* total solids, *TN* total nitrogen, *BOD* biological oxygen demand, *COD* chemical oxygen demand, *NO*_*3*_ nitrate, *NH*_*4*_ ammonium, *PO*_*4*_ phosphate

### Isolation of oleaginous yeast isolates from SWW

Eight yeast isolates with distinctive morphology were isolated from SWW by bio-trap enrichment and direct dilution on YEME medium. Among these, five isolates (ASEY1, ASEY2, ASEY3, ASEY4, and ASEY5) were from the enriched samples (wastewater dumped soil), and three (ASY1, ASY2, and ASY3) were from the SWW samples. Morphological characterization showed that the isolates are ellipsoidal, occurring as a single cell or as parental bud pairs. After 1–2 days of incubation at 30 °C, the cultures turned creamy white to pure white. Even in older cultures, no hyphae were noticed. Cultures grew better at 28–30 °C than at 31–35 °C.

### Primary screening

#### Amylase activity

**Fig. 1:**
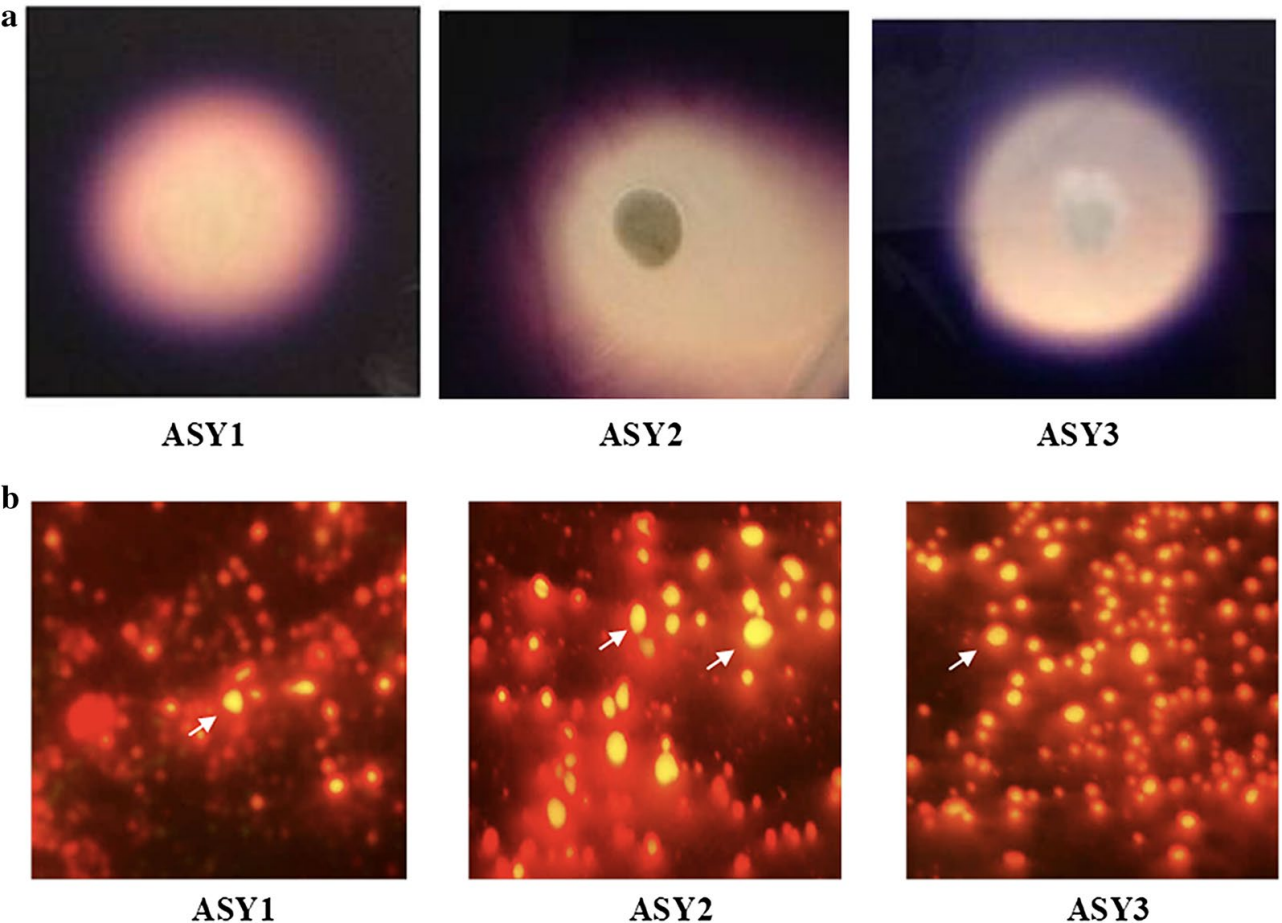
**a** Amylase activity of the yeast isolates from SWW in starch agar medium **b** Fluorescence microscopic images of Nile red-stained yeast isolates from SWW

As SWW is rich in starch, we screened the isolates for amylase activity. Three (ASY1 (7 mm), ASY2 (15 mm), and ASY3 (10 mm)) of the eight yeast isolates were positive for amylase activity indicated by a clear zone around the individual colonies in starch agar (Fig. 1a). The yeast isolate ASY2 showed the maximum zone of hydrolysis (15 mm).

#### Lipid detection by fluorescence microscopy

The accumulated lipids or SCOs get deposited as intracellular lipid bodies, which can easily be detected by a fluorescent probe, such as Nile red. All three amylase-positive isolates (ASY1, ASY2, and ASY3) were selected for fluorescence analysis. Surprisingly, all three isolates showed the presence of lipid globules as appearing yellow-colored cells (Fig. 1b). Fluorescence microscopy revealed oval and ellipsoidal lipid bodies throughout the cell in the yeast isolate ASY2.

### Secondary screening of yeast isolates in synthetic medium and SWW

**Fig. 2:**
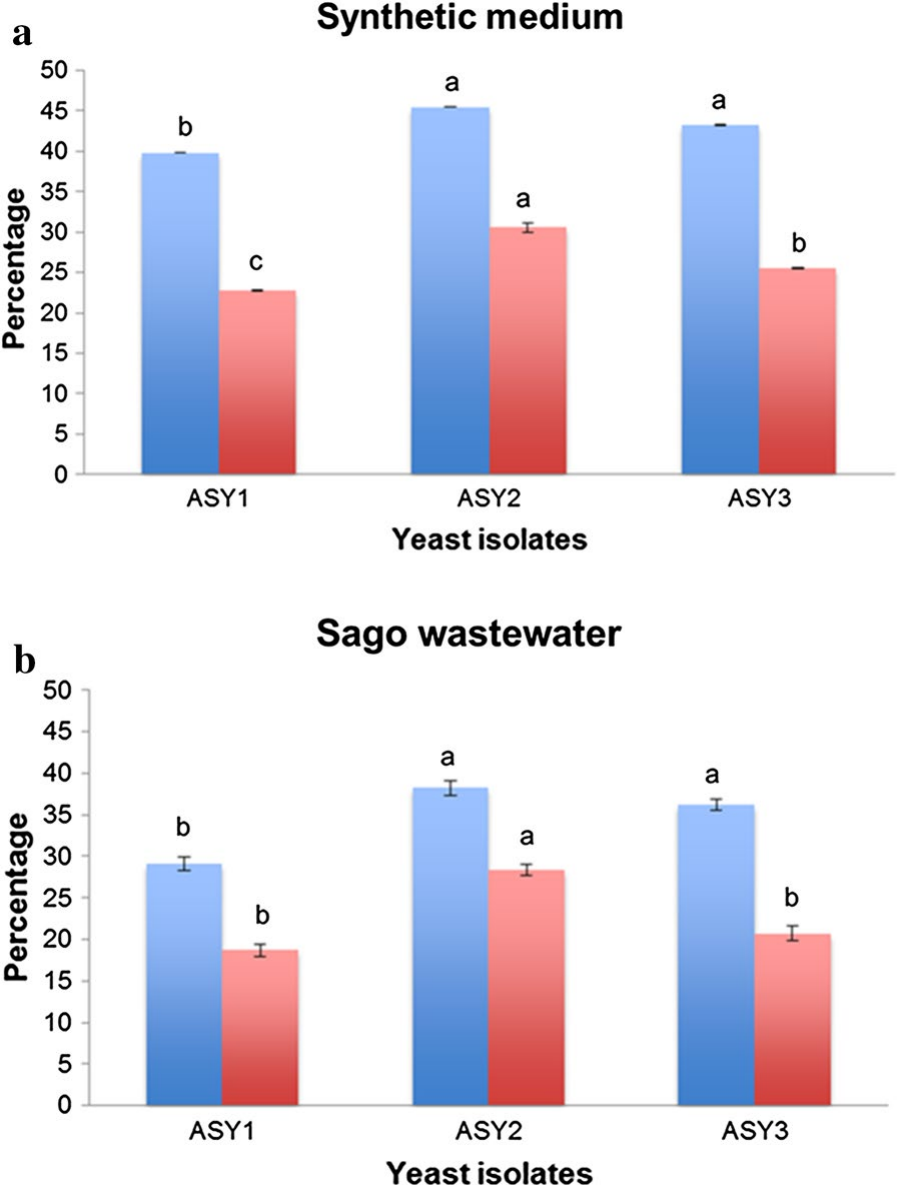
**a** Lipid accumulation and starch utilization by yeast isolates in synthetic medium. **b** Lipid accumulation and starch utilization by yeast isolates in sago wastewater. The same alphabet letter represented in the top of the bar is not significantly different from each other at p ≤ 0.05 according to Duncan’s multiple range test (DMRT)

The yeast isolates (ASY1, ASY2, and ASY3) were grown in high carbon and nitrogen-limited lipid production medium to induce lipid synthesis. The biomass yield (g L^−1^), lipid yield (g L^−1^), and lipid content (%) were monitored at 24-h intervals (30 °C and pH 6.0). At 72 h, the yeast isolate ASY2 recorded significantly higher biomass (6.21 g L^−1^), lipid yield (2.82 g L^−1^), lipid content (45%), and starch utilization (30.46%) (*p* < 0.05) than other isolates. The lipid content (41.54%) was comparable with the yeast *Cystobasidium oligophagum* JRC1 [20] and was lesser than *Cryptococcus terricola* JCM 2452 (61.96%) in batch culture [21]. Lipid content and starch utilization of the three isolates in synthetic medium and SWW, respectively, are shown in Fig. 2a, b.

The three yeast isolates were grown in SWW at a starch concentration of 10 g L^−1^ at pH 6.0, temperature 30 °C, and 150 rpm, in an incubator shaker. Like the synthetic medium, ASY2 recorded significantly maximum biomass (0.1473 g L^−1^), lipid yield (0.056 g L^−1^), and lipid content (38.01%) in SWW at 72 h of incubation. Regarding starch utilization, a significant difference was observed between the yeast isolates, in which the isolate ASY2 recorded maximum lipid (28.39%). Xue et al. [22] observed a lipid content of 20% in the oleaginous yeast *Rhodotorula glutinis* cultured on monosodium glutamate wastewater with the addition of 15 g L^−1^ of glucose at 72 h. Saenge et al. [23] used palm oil mill effluent for lipid production and obtained a lipid content of 20.97%.

### Confirming oleaginicity by flow cytometry

In order to confirm the oleaginicity of the yeast isolate ASY2, flow cytometry was carried out in comparison with the non-oleaginous yeast strain *Saccharomyces cerevisiae.* Nile red has been extensively used as a lipid probe to detect intracellular lipids in intact cells by fluorescence microscopy and flow cytometry. Nile red fluorescence signals of both forward (25,584.84) and side scatter (granularity) (15,663.90) obtained from ASY2 were found to be higher compared to the fluorescence signals of forward (4602.63) and side scatters (2368.44) from the non-oleaginous yeast strain *S. cerevisiae*. The side scatters (granularity) is one of the main parameters and can be used to detect intracellular lipids in yeast strains. The results confirmed that the yeast isolate ASY2 possessed high intracellular lipid accumulating potential and is oleaginous.

### Identification and characterization of the yeast isolate

**Fig. 3:**
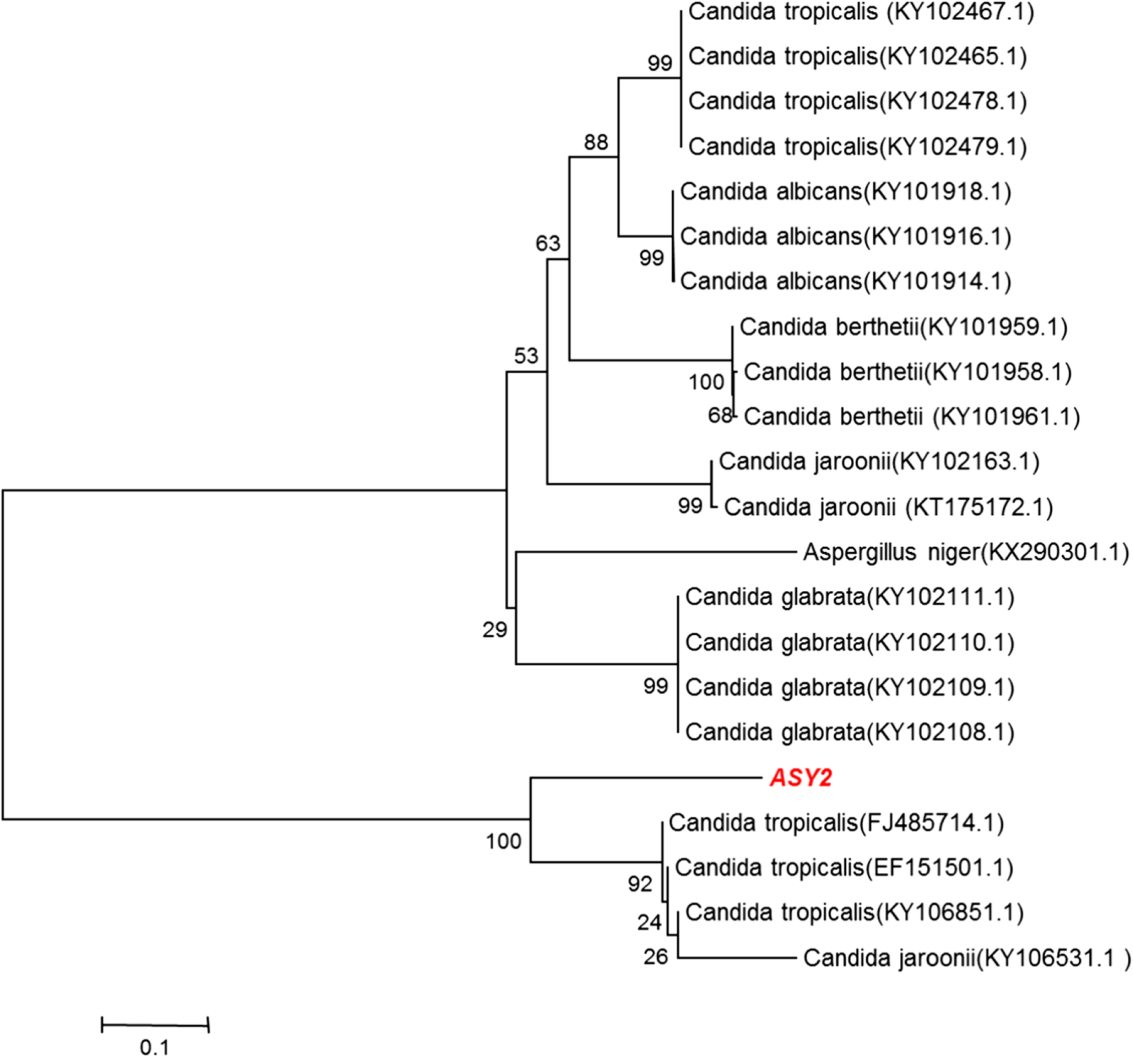
Phylogenetic tree constructed using the sequence homology of NL region of the yeast isolate using the neighbor-joining method

The yeast isolate ASY2 was identified on the basis of 28S rRNA sequence homology as analyzed using the universal primers of NL1 and NL4. The samples showed amplicon bands of size 600 bp. The analysis of ASY2 sequence using BLAST showed 100% similarity with *Candida tropicalis* (Accession No. MH011502). The sequence data of the above strain is available in the NCBI database. Phylogenetic analysis was done using the neighbor-joining method and the yeast strain was positioned and clustered with their corresponding sequence-matching genomes (Fig. 3).

### Growth and lipid production kinetics of *C. tropicalis* ASY2 grown in SWW

**Fig. 4:**
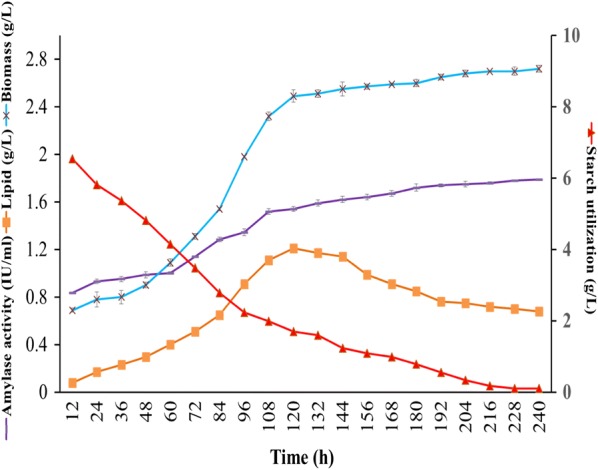
Kinetics of lipid and biomass production, starch utilization, and amylase activity obtained during the cultivation of *C. tropicalis* ASY2 in SWW

The yeast strain *C. tropicalis* ASY2 was grown for 10 days (pH 6, temperature 30 °C, and 150 rpm) in SWW with a C:N ratio of 10:1 and the samples were withdrawn at 12-h intervals up to 240 h of fermentation. The biomass yield, lipid yield, and lipid content reached maximum (2.49 g L^−1^, 1.21 g L^−1^, and 48.59%, respectively) at 120 h of incubation. These are the highest values reported so far, using wastewater as the sole substrate for lipid production by oleaginous yeast strains (Table 2). The lipid content and yield declined slightly after 120 h and stabilized at 240 h (Fig. 4). Most oleaginous yeasts accumulate lipid during the post-stationary phase and an incubation period of 120–192 h is favorable for achieving the highest lipid content that varies among different yeasts [20]. Xue et al. [24] obtained a maximum lipid content of 35% in the oleaginous yeast *Rhodotorula glutinis* cultivated on cornstarch wastewater supplemented with waste syrup.

**Table 2:** Comparative evaluations of different industrial wastewaters for the lipid production by oleaginous yeasts

Wastewater type	Oleaginous yeast	Biomass yield (g L^-^^1^), lipid yield (g L^-^^1^), lipid content (%)	Lipid productivity (g L^−1^ day^−1^), incubation period (days)	Nutrient removal efficiency	References
Monosodium glutamate wastewater	*Rhodotorula glutinis*	2.44, 0.2, 9.04	0.04, 5	85.51% COD	[28]
Monosodium glutamate wastewater + 15 g L^−1^ glucose	*Rhodotorula glutinis*	25, 5, 20	1.67, 3	45% COD	[22]
Fishmeal wastewater	*Lipomyces starkeyi* HL	5.34, 1.11, 20.80	0.185, 6	81.5% COD	[26]
Fishmeal wastewater + 20 g L^−1^ glucose	*Lipomyces starkeyi* HL	17.6, 2.7, 15.3	0.45, 6	43.4% COD	
Palm oil mill effluent	*R. glutinis* TISTR 5159	4.15, 0.87, 20.97	0.29, 3	40.50% COD	[23]
Palm oil mill effluent (two fold diluted)	*Yarrow lipolytica*	2.78, 1.69, 61	0.56, 3 + 3 (two phase)	–	[29]
Wastewater after butanol fermentation	*Trichosporon dermatis*	7.4, 0.99, 13.5	0.2, 5	68% COD	[30]
Pulp and paper industry effluent + 70 g L^−1^glucose	*R. kratochvilovae* HIMPA1	13.87, 8.56, 61.71	1.43, 6	77.36% BOD	[31]
Cattle livestock wastewater	*Candida pseudolambica*	2.19, 0.77, 35.3	–	–	[27]
	*Issatchenkia occidentalis*	6.54, 1.89, 28.9	–	–	
Sago processing wastewater (SWW)	*Candida tropicalis* ASY2	2.49, 1.21, 48.59	0.24, 5	83.52% COD; 92.11% BOD; 78.94% cyanide degradation	This study

Chi et al. [25] used food and municipal wastes for microbial lipid production and attained 18–26% lipid content by co-culturing yeast and algae. Huang et al. [26] used saline and organic-rich fishmeal wastewater for lipid production by *Lipomyces starkeyi* HL and achieved 20.8% without any supplement. Chung et al. [27] used hydrodynamic cavitation pretreated livestock wastewater for biodiesel production by *Candida pseudolambica* and *Issatchenkia occidentalis* and recorded a lipid content of 35.3% and 28.9%, respectively. These results indicated that higher lipid yield (48.59%) was achieved without an external nutritional source supplement in SWW in a period of 120 h. These findings provide scope to enhance the lipid content by adding exogenous nutrients and other additives.

### Starch utilization and amylase secretion by *C. tropicalis* ASY2 in SWW

In SWW, the starch consumption by ASY2 commenced from the first day with a biomass and lipid concentration of 0.69 g L^−1^ and 0.08 g L^−1^, respectively, by consuming 35% (3.45 g L^−1^) of starch from SWW (Fig. 4). The maximum lipid content (48.59%) was obtained at 120 h of fermentation by consuming 83.89% (8.3 g) of starch with a mean biomass of 2.49 g L^−1^. Thereafter, the yeast biomass increased steadily up to 240 h from 2.49 to 2.72 g L^−1^ with a gradual decline in lipid yield. At the end of fermentation (240 h), the remaining starch (1.7 g L^−1^) was almost utilized by the yeast strain. For obtaining a maximum lipid yield of 1.21 g L^−1^, 8.3 g of starch was utilized. The conversion rate or Y_L/C_ (g of lipid produced per g of substrate consumed) ranged from 0.079 to 0.135 g of lipid/g of starch consumed. The highest Y_L/C_ value (0.135 g g^−1^ of starch utilized) was obtained at 120 h of incubation, which suggests that higher Y_L/C_ values exhibit higher substrate conversion efficiency of the yeast strain.

Oleaginous yeasts *Yarrowia lipolytica, Candida* sp., and *Rhodotorula glutinis* recorded Y_L/C_ values in the range of 0.11–0.22 g of lipids per gram of glucose consumed [32]. Oleaginous yeast *Torulaspora maleeae* Y30 grown on pure glucose had a conversion yield of 0.034–0.059 g of lipids with glucose concentration varying from 40 to 100% [33]. Papanikolaou and Aggelis [34] reported maximum lipid conversion efficiency with glycerol (0.30 g L^−1^) compared to pure glucose. Therefore, the maximum lipid conversion efficiency (Y_L/C_) of 0.135 using SWW as a substrate signifies its amenability as a low-cost feedstock for biodiesel production. Meanwhile, the amylolytic activity was gradually increased and attained 1.79 IU mL^−1^ at the end of fermentation.

### FTIR analysis of the yeast biomass and extracted lipid from SWW

**Fig. 5:**
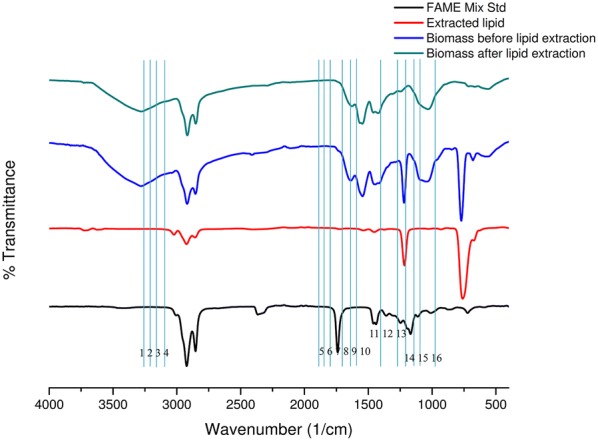
FTIR analysis of yeast biomass before and after lipid extraction and extracted lipid in SWW

Transmission spectra were acquired for the extracted lipids, FAME mix, and yeast biomass before/after lipid extraction at 120 h of incubation when the highest lipid production was recorded. The peaks assigned to the corresponding functional groups [35] are presented in Table 3 and Fig. 5. The absence of peaks from 4000 to 3010 cm^−1^ region in the extracted lipid indicated the absence of free hydroxyl group (–OH) and an amine group (-NH_2_). The spectral regions responsible for lipids are 3020–2800 cm^−1^, 1800–1700 cm^−1^, 1500–1300 cm^−1^, 1100–1200 cm^−1^, and 800–700 cm^−1^; for proteins are 800–700 cm^−1^; and for phospholipids and polyphosphates are 1300–1200 cm^−1^. The peaks at 3010 cm^−1^, 2955 cm^−1^, 2925 cm^−1^, and 2850 cm^−1^ indicated asymmetric stretching of–CH_2_/CH_3_ acyl chains, while peaks recorded at 1745 cm^−1^ indicated C=O stretching. The peaks observed at 1465 cm^−1^ and 725 cm^−1^ disclosed –CH_2_ deformation. The peak at 1155 cm^−1^ indicated C–O–C stretching of the lipids. Peaks corresponding to the proteins (amide I, II, III band) were observed for yeast biomass at wavenumbers 1650 cm^−1^, 1579 cm^−1^, and 1410 cm^−1^. The wavenumbers at 1265 cm^−1^, 1080 cm^−1^, 875 cm^−1^, and 985 cm^−1^ indicated the presence of polyphosphate, phospholipid, and carbohydrates, respectively, in yeast biomass.

**Table 3:** Peak assignment in the FTIR spectra for the lipid extract of *C. tropicalis* ASY2

Peak no.	Wavenumber (cm^−1^)	Peak assignment	Main biomolecules
1	3010	=C–H stretching	Lipid
2	2955	C–H asymmetric stretching of –CH_3_	Lipid
3	2925	Stretching of >CH_2_ of acyl chains (asymmetric)	Lipid
4	2850	Stretching of CH_2_ of acyl chains (symmetric)	Lipid
5	1745	C=O stretching	Lipid
6	1680–1640	Amide I band (C=O stretching)	Protein
7	1580–1520	Amide II (CONH bending)	Protein
8	1465	CH_2_ deformation	Lipid
9	1410	Amide III band (C–N stretching)	Protein
10	1380	CH_3_ bending	Lipid
11	1240 –1265	P=O stretching (asymmetric) of >PO_2_ phosphodiesters	Polyphosphate, phospholipid
12	1155	C–O–C stretching	Lipid
13	1080	P=O stretching (symmetric) of >PO_2_	Polyphosphate, phospholipid
14	900–1200	C–O and C–C stretching, C–O–H and C–O–C deformation	Carbohydrate
15	875	P–O–P stretching	Polyphosphate, phospholipid
16	725	CH_2_ deformation	Lipid

### FAME analysis for the lipid produced by the yeast

The GC-FID analysis was done for the yeast biomass with high lipid accumulation (120 h of incubation). The FAME composition obtained for the lipid from *C. tropicalis* ASY2 was compared with the fatty acid composition of soybean and jatropha oils to evaluate its suitability as feedstock for biodiesel production [36]. We observed significantly more oleic acid content (41.33%) than that of jatropha (39.08%) and soybean oils (31.14%) (Table 4).

**Table 4:** Comparative analyses of fatty acids of *C. tropicalis* ASY2 with crude soybean and jatropha oil

Type of fatty acids	Soybean oil (%)	Jatropha oil (%)	*C. tropicalis* ASY2 (%)
C16:0 (palmitic acid)	9.88	14.66	0.01
C16:1 (palmitoleic acid)	–	0.94	0.07
C18:0 (stearic acid)	5.06	6.86	0.18
C18:1 (oleic acid)	31.14	39.08	41.33
C18:2 (linoleic acid)	52.77	32.48	1.70
C18:3 (α-linolenic acid)	–	–	3.48
C18:3 (γ-linolenic acid)	1.14	0.30	3.1
C20:1 (arachidic acid)	–	–	11.61
C24:1 (nervonic acid	–	–	9.72
Saturated fatty acid (SFA)	–	–	26.99
Unsaturated fatty acid (MUFA + PUFA)	–	–	59.77

Approximately 3.48% alpha-linolenic acid was detected, which is absent in jatropha and soybean oils. The gamma-linolenic acid content of ASY2 (3.1%) was more than that of soybean oil (1.14%). Compared to other oils like jatropha oil (21.52%), sunflower oil (4.5%), soybean oil (15%), and rapeseed oil (6.6%), the overall saturated fatty acid (SFA) was high (26.99%); however, it was less than that recorded in palm oil (44.41%). Some significant biodiesel characteristics like oxidative stability, kinematic viscosity, saponification value, iodine value, and cetane number depend on the saturation degree and length of the fatty acid chain [37]. Earlier studies have proven that a lower saturation degree is safer for kinematic viscosity and melting point [20]. Monounsaturated fatty acids (MUFA, 47.89%) and polyunsaturated fatty acids (PUFA, 11.88%) were also detected. The FAME data demonstrated that the lipids obtained from *C. tropicalis* ASY2 could be used to produce biodiesel with suitable fuel characteristics. These findings indicate that it can be an alternative option for the production of biodiesel.

### Decontamination parameters

#### Nutrient, COD, and BOD removal from SWW in relation to lipid production

**Fig. 6:**
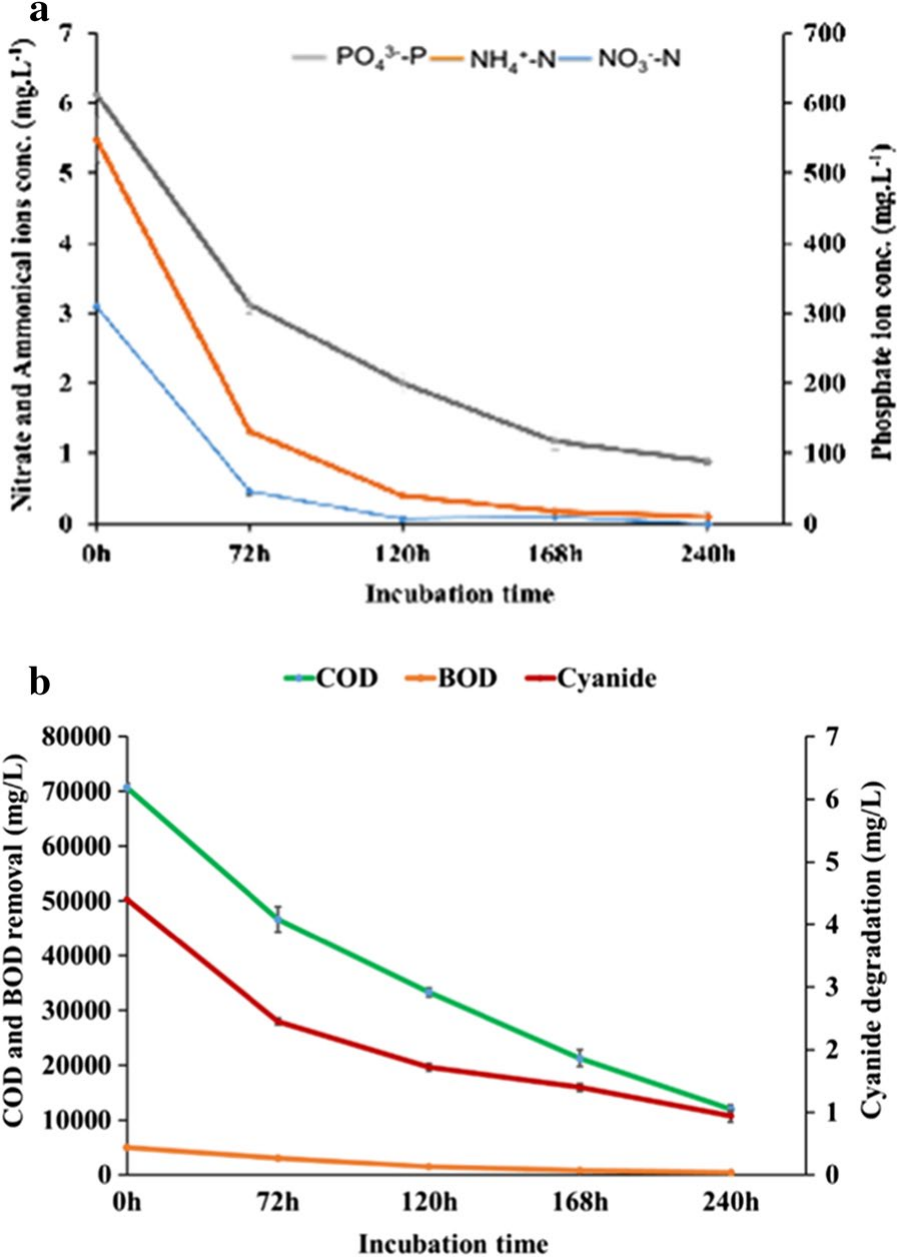
**a** Depletion of ammoniacal (NH_4_^+^-N), nitrate (NO_3_^−^-N) and phosphate ions (PO_4_^3^-P) in SWW by *C. tropicalis* ASY2. **b** COD, BOD reduction and cyanide degradation in SWW by *C. tropicalis* ASY2

The initial content of NO_3_, NH_4_, and PO_4_ ions in SWW was 3.10, 5.48, and 611.67 mg L^−1^, respectively. At 120 h of incubation, where maximum lipid accumulation was observed in the yeast strain, most of NO_3_ (0.07 mg L^−1^) and NH_4_ ions (0.40 mg L^−1^) in SWW got exhausted, and PO_4_ ions reduced to a greater extent (200.33 mg L^−1^). Over 240 h of the fermentation period, the yeast strain *C. tropicalis* ASY2 utilized almost 100% of NO_3_, 98.17% of NH_4_, and 85.45% of PO_4_ from SWW (Fig. 6a).

Wynn et al. [38] observed a change from average growth to lipid accumulation in *Mucor circinelloides* and *Mortierella alpina* batch cultures under nitrogen-limited conditions. A decrease in intracellular NH_4_ ions inhibits phosphofructokinase enzyme activity leading to citrate accumulation, which is the source of acetyl-CoA for lipid production. Dourou et al. [39] reported a decrease in lipid production rate due to a decrease in malic enzyme activity, which is the rate-limiting enzyme in lipid production pathway. Malic enzyme activity can only be restored by de novo synthesis, which requires an N-source. In the present study, the decreased lipid concentration and lipid content of yeast after 120 h of cultivation in SWW (even though the biomass yields remain unchanged) might be due to the degradation of malic enzyme, which could not be re-synthesized owing to the exhaustion of N-source.

In addition to nutrient removal, the time course of COD and BOD removal from SWW revealed that the initial soluble COD (70.67 g L^−1^) and BOD (5.04 g L^−1^) concentrations in SWW decreased to 12 g L^−1^ and 0.4 g L^−1^, respectively, throughout 240 h (Fig. 6b). The maximum removal of COD and BOD (83.52% and 92.11%, respectively) was achieved at the end of fermentation.

#### Biodegradation of cyanide in SWW

The rate of cyanide removal was increased over time and the maximum degradation of 78.94% was achieved at 240 h of cultivation in SWW (the initial cyanide content was 4.46 mg L^−1^). We observed the final cyanide content of 0.94 mg L^−1^ in the treated wastewater (Fig. 6b). The reason behind the cyanide degradation is the presence of enzymes such as cyanide hydratase, nitrile hydratase, thiocyanate hydrolase, nitrilase and cyanidase in oleaginous yeast *C. tropicalis* ASY2. The enzyme present in yeast utilizes cyanide as a source of carbon and nitrogen for growth [40]. For cyanide removal, the current wastewater treatment employs physical and chemical methods, which are often expensive and involves the use of additional hazardous reagents (chlorine and sodium hypochlorite) [41]. But in the present study, we employed the microbial system of cyanide removal, which was a cost-effective and efficient method.

#### Other physicochemical parameters

We further estimated various physicochemical parameters of treated SWW, such as pH, EC, TDS, TS, salinity, and starch. The pH of untreated SWW (4.67) was adjusted to 6.0 to promote yeast growth. We detected a slight increase in pH (6.7) of the treated wastewater. EC of the treated SWW was reduced to 4.20 dS m^−1^ from 6.30 dS m^−1^. The raw wastewater initially contained about 4573 mg L^−1^ of insoluble starch and fibers that were reduced to 1260 mg L^−1^ in treated wastewater. There was 52.01% and 49.47% decrease in the salinity and TDS, respectively.

## Conclusion

In this research, we developed a novel process for microbial oil production from sago processing wastewater (SWW) with concurrent removal of pollutants using oleaginous yeast. SWW served as an excellent substrate for oleaginous yeast *C. tropicalis* ASY2 and resulted in the maximum lipid production (0.010 g L^−1^ h^−1^, 49% lipid content) suggesting that SWW could be a zero-cost feedstock for biodiesel production. Furthermore, the FAME profile of yeast biodiesel revealed higher oleic acid content of 41.33% compared to jatropha and soybean oils, suggesting the superiority of biodiesel produced from SWW. Interestingly, the simultaneous decontamination potential of the strain ASY2 (79% cyanide degradation, 84% COD and 92% BOD removal) paves the way for the reusability of wastewater for irrigation purposes. Thus, using SWW and oleaginous yeast strain proves to be a cost-effective and eco-friendly method for biodiesel production with simultaneous wastewater treatment for a sustainable environment. Any further advancement towards scale-up and its optimization would be the way forward towards a greener and sustainable development of the nation.

## Methods

### Chemicals and reagents

All materials for culture medium and chemicals, such as n-hexane, methanol, chloroform, picric acid, sodium carbonate, and Nile red were bought from Hi-Media Laboratories (Mumbai, India). PCR primers and FAME standards were purchased from Sigma-Aldrich (St Louis, MO, USA).

### Sago processing wastewater collection

Sago processing wastewater (SWW) was collected from M/S. Sri Selliamman Sago Factory located in the Namakkal district of Tamil Nadu, India. The collected SWW was taken in an airtight container and stored at 4 °C for further analysis. The physicochemical properties of SWW were analyzed following the standard method of water and wastewater analysis [42]. The depletion of macronutrients, such as nitrogen (ammoniacal and nitrate) and phosphorus in the wastewater was estimated using probe photometer kits (Palintest 7500 Photometer, USA) according to the manufacturer’s protocol.

### Isolation and screening of oleaginous yeast strains

Biotrap enrichment method, direct dilution, and plating process were used to isolate the oleaginous microbes. In the bio-trap enrichment method, the substrates namely ‘thippi’ and starch were filled one-third in 15-mL perforated falcon tubes and kept in wastewater discharge pipeline for enrichment. After 15 days, the enriched samples were serially diluted (10^−3^ to 10^−5^ fold dilution). Aliquots of 1 mL from the final dilutions were plated on yeast media (yeast extract mannitol agar) and incubated at room temperature (30 ± 2 °C) for 3–5 days. A single distinct colony from each plate was isolated and purified. The quadrant streak method was used for purification. The cultures were maintained in agar slants at 4 °C.

### Primary screening of oleaginous yeasts

#### Starch hydrolysis

Starch agar (starch, 1 g; peptone, 0.5 g; yeast extract, 0.3 g; NaCl, 0.3 g; agar, 20 g L^−1^) was used to test the amylase activity of the isolates [43]. The yeast isolates were grown in starch agar for 3–5 days and incubated at 30 °C. The plates were flooded with 1% iodine solution and observed for a clear zone formed by starch hydrolysis around the yeast colony.

#### Lipid fluorescence

All amylase-positive yeast isolates were evaluated for intracellular lipid bodies by Nile red fluorescence staining [44]. Nile red (9-diethylamino-5-benzo (a)phenoxazinone) is a red phenoxazine dye, which selectively stains lipophilic substances. An aliquot of 10 μL of Nile red (0.1 mg mL^−1^) was mixed with 100 μL of the individual culture suspension derived from the carbon-rich medium. Phosphate buffer saline (2 mL) was added and the tubes kept for 5 min in the dark. The lipid bodies were visualized under a Nikon Eclipse 80i light microscope (Nikon Instruments, Tokyo, Japan) equipped with a digital camera (465–495 nm excitation filter, 505 nm diachronic mirror, and 515–535 nm barrier filter).

### Secondary screening of yeast isolates

The three positive isolates from primary screening were further evaluated for their intracellular lipid accumulation in both synthetic medium and SWW. The isolates were grown in high carbon and low nitrogen synthetic medium with C:N ratio of 10:1 (composition per liter: starch, 10 g; ammonium sulfate, 0.5 g; potassium dihydrogen phosphate, 7 g; disodium hydrogen phosphate, 2.5 g; magnesium sulfate, 1.5 g; ferric chloride, 0.15 g; calcium chloride, 0.15 g; zinc sulfate, 0.02 g; and manganese sulfate, 0.06 g; pH = 6) for 3 days at 30 °C in an incubator shaker (150 rpm).

The isolates were also grown in SWW under similar conditions after adjusting the initial pH (4.67 to 6) with an alkaline solution (NaOH). The starch content in SWW varied (7 to 10 g L^−1^) based on crushing and extraction. Hence, the initial starch concentration of SWW was adjusted to 10 g L^−1^. Both synthetic medium and SWW were inoculated at 10^6^ cells mL^−1^ density and the samples along with yeast biomass were withdrawn at regular intervals and analyzed for different lipid production parameters.

### Determination of oleaginicity by flow cytometry

The oleaginicity of the yeast with the highest lipid accumulation was compared with one of the well-known non-oleaginous yeast strain *S. cerevisiae* (Baker’s yeast) by flow cytometry. Approximately 10^6^ cells mL^−1^ were inoculated into yeast extract malt extract (YEME) broth and incubated under shaking condition (150 rpm) for 3 days. After incubation, the yeast cells were centrifuged at 6000 rpm for 3 min, and the pellets were washed with phosphate buffer. This step was repeated twice and the pellets were suspended in phosphate buffer, and the cell concentrations were determined. A known intensity of cells was stained with Nile red (Sigma-Aldrich, St Louis, MO, USA) for the lipid analysis and was incubated for 5 min in the dark at room temperature. After calibrating the flow cytometer C6 Accuri (BD Biosciences, USA) using six peak validation beads (BD Biosciences, USA), the samples were injected into the sampling port and analyzed using the BD sampler workspace. A total of 10,000 cells were measured for each sample at a slow fluidic rate (excitation wavelength: 488 nm, emission wavelength: FL2 channel 585 nm ± 40 nm, flow rate 14 μL/min, 10 μm core size).

### Identification of the oleaginous yeast isolate by rRNA sequencing and phylogenetic analysis

The oleaginous yeast isolate with high biomass, lipid productivity, and lipid content was identified by 28S rRNA sequencing. The genomic DNA was extracted using Bust n’ Grab method from the isolate grown for 3 days in YEME. The concentration and purity of the DNA were estimated by Nanodrop spectrophotometer and agarose gel electrophoresis. The 28S rRNA gene was amplified by polymerase chain reaction (PCR) using a master cycler gradient thermocycler (Eppendorf, Germany). PCR was performed in a volume of 25 μL containing 1 μL of DNA, 12 μL of PCR Master Mix (Applied Biosystems, UK), 2 μL each of forward and reverse primers, and 8 μL of PCR water (Sigma, St Louis, MO, USA).

To amplify 28S rRNA, NL1 (5′-GCATTCAATAAGCGGAGGAAAAG-3′) and NL4 (5′-GGTCCGTGTTTCAAGACGG-3′) primers were used with the following PCR conditions: an initial denaturation at 95 °C for 5 min; 40 cycles of 94 °C for 40 s (denaturation), 55 °C for 1 min (annealing), and 72 °C for 1 min (primer extension); and a final extension at 72 °C for 10 min. The amplicons were resolved by electrophoresis on 1.5% agarose gels, and the gels were visualized and documented using a BioRad Gel imaging system (BioRad, USA). The PCR products were purified using spin columns (Qiagen, Germany) and sequenced by automated sequencing (Euro Fins Biotech Pvt. Ltd., Germany).

#### Phylogenetic analysis

The identity of the isolate, ASY2 was identified using nucleotide BLAST (using NCBI database), assuming at least 99% identity as the best hit, and the obtained sequence was submitted to GenBank. Taxonomic positioning and phylogenetic tree construction were done using the neighbor-joining method of Saitou and Nei [45] with MEGA 4.0 software along with the existing 28S rRNA gene sequences of related eukaryotes obtained from NCBI GenBank database.

### Kinetic study of the selected yeast isolate for the production of lipid

The yeast strain ASY2 was inoculated at a density of 10^6^ cells mL^−1^ in SWW (pH set to 6) and cultivated for 10 days at 30 °C in an incubator shaker (150 rpm). The shaking speed limit used for different yeast strains such as *Cystobasidium oligophagum* JRC1: 120 rpm, *Rhodococcus opacus*: 120 rpm, *R. opacus*: 250 rpm under batch mode cultivation were referred from earlier works [20, 46, 47]. Periodically, the SWW samples, along with yeast biomass, were withdrawn and analyzed for various parameters related to lipid production and decontamination of SWW.

### Microbial lipid production process parameters

#### Biomass yield

The dry weight of the yeast was determined gravimetrically as follows: 50 mL of culture was centrifuged at 6000 rpm for 10 min, and the biomass pellet was washed twice with sterile distilled water and dried in a hot air oven at 40 °C until a constant weight was obtained (usually 24 h).

#### Lipid content

The yeast biomass (100 mg) was homogenized with 20 volumes of chloroform:methanol mixture (2:1; v:v) and agitated overnight in an orbital shaker at room temperature. The lipid phase was separated by adding 0.2 volume of distilled water and centrifuged at 3000 rpm for 10 min. The upper phase was siphoned out, and the lower chloroform phase containing lipids was evaporated under vacuum. The extracted lipids were measured gravimetrically [48].

#### Residual glucose

Residual glucose in SWW was analyzed using the DNS method [49]. Of the supernatant, 1 mL was added into a 15-mL glass tube and the sample volume was made to 3 mL by adding distilled water. Further, 3 mL of DNS reagent was added to the tubes and placed in a water bath at 90 °C for 5 min. When the contents were still warm, 1 mL of 40% Rochelle salt solution (potassium sodium tartrate) was added to the tubes. The tubes were then cooled down to ambient temperature, and the color intensity (dark red) was read at a wavelength of 510 nm using a spectrophotometer against distilled water (1 mL) as blank. The procedure mentioned above was repeated for all SWW samples. The final concentration of glucose was determined by a standard curve made by using 0–500 μg of glucose.

#### Residual starch

Residual starch in SWW was analyzed using the phenol–sulfuric acid method [50]. The supernatant sample (0.1 mL) was added into a 15-mL glass tube and the volume was made up to 1 mL with distilled water. Into the tube, 1 mL of phenol and 5 mL of 96% sulfuric acid were added. The contents were mixed well by inverting the tubes several times and left undisturbed for 10 min. The tubes were shaken and placed in a water bath at 25–30 °C for 20 min. The contents were cooled down to ambient temperature, and the color intensity was read at a wavelength of 490 nm using a spectrophotometer against distilled water (1 mL) as blank. The final concentration of starch was determined by a standard curve made by using 0–500 μg of glucose.

### Fourier transform-infrared spectroscopy (FTIR) analysis

The spectra of biomass and lipid samples of *C. tropicalis* ASY2 were obtained using an FTIR (FTIR–6800 JASCO, Japan). The absorbance spectra were recorded in a wavenumber range of 4000 to 400 cm^−1^ with a spectral resolution of 4 cm^−1^ and 32 scans per sample.

### Gas chromatography analysis of FAME

Fatty acid methyl esters (FAME) were extracted from *C. tropicalis* ASY2 to identify and quantify the fatty acid composition by direct transesterification method. The dried yeast cells (100 mg) were added to a mixture of 10 mL of 0.1% methanol sulfuric acid, mixed vigorously, and then mixed with 10 mL of chloroform. The mixture was heated at 80 °C for 2 h and then cooled down to ambient temperature. Distilled water (1 mL) was added and the suspension was centrifuged at 1500 rpm for 5 min for the phase separation and also for removing water-soluble impurities. At the same time, the water residue from the organic phase (FAME) was removed by adding anhydrous Na_2_SO_4_ [51]. The lower phase containing FAME was analyzed by gas chromatography (Perkin Elmer Clarus 680, USA) with flame ionization detector (FID) and Elite-5 column (30 m × 0.25 mm × 0.25 µm). The injection temperature was 220 °C; the initial column temperature was 160 °C that attained a final temperature of 190 °C at a rate of 3 °C min^−1^; the detector temperature was 270 °C. The carrier gas (helium) was maintained at a flow rate of 1.3 mL min^−1^. The FAME composition of lipids was determined by comparing the retention time and sample peak area with standard FAME mix (FAME mix C4-C24, catalog No: LC16860V, Sigma):

$${\text{FAME compostion}}, \% = \left( {\frac{{{\text{Standard conc}}.}}{{{\text{Standard area}} }} \times \frac{\text{Sample area}}{{{\text{Sample wt }}\left( {\text{mg}} \right)}} \times {\text{Dilution factor}}} \right)/ 10,000,$$

$${\text{FAME content}} = \frac{{{\text{FAME }}\left( {\text{mg}} \right)}}{{{\text{Neutral lipid }}\left( {\text{mg}} \right)}} \times 100.$$

### Sequestration of pollutants

Yeast biomass was completely removed either by centrifugation or filtration from SWW and the physicochemical characteristics of the decontaminated SWW samples were analyzed as described earlier to determine the pollutant sequestration capability of the strain.

### Biological oxygen demand

Biological or biochemical oxygen demand (BOD) is the measure of organic load in the sago effluent sample. The samples were diluted with water and filled in two sets of BOD bottles up to the brim without entrapment of air. One set was measured for initial dissolved oxygen (DO) immediately and the other set was incubated in BOD incubator at a temperature of 25 ± 2 °C for 5 days, the DO content in these bottles was measured as final. The BOD of wastewater was calculated according to the formula given,

$${\text{BOD}}_{ 5} {\text{mg}}/{\text{l }} = \, \left( {{\text{DOI}}_{\text{S}} {-}{\text{ DOF}}_{\text{S}} } \right) - \left( {{\text{DOI}}_{\text{B}} {-}{\text{ DOF}}_{\text{B}} } \right) \times \frac{{V_{t} }}{{V_{s} }},$$

where $V_{\text{s}}$ = volume of sample in mL, $V_{\text{t}}$ = titre value of thiosulfate in mL, DOI_S_ – DOF_S_ = initial DO of sample and final DO of sample, DOI_B_ – DOF_B_ = initial DO of blank and final DO of blank.

The difference between initial and final DO was measured as BOD and the results were reported as BOD_5_ mg L^−1^.

### Chemical oxygen demand

Chemical oxygen demand (COD) is the oxygen requirement of a water sample for the oxidation of organic and inorganic matter. The COD of SWW was determined by following the open reflux method [52]. Of the sample, 20 mL was added in the reflux unit flask, and then 10 mL of potassium dichromate solution, a pinch of each silver sulfate and mercuric sulfate and 30 mL of sulfuric acid were added. Liebig condenser was attached to the mouth of the flask on a hot water bath or heating mantle for at least 2 h to reflux the contents. Then, the flask was cooled, detached from the unit and its contents diluted to about 150 mL by adding distilled water. Then 2–3 drops of ferroin indicator (695 mg ferrous sulfate and 1.485 g of 1,10-phenanthroline monohydrate together dissolved in distilled water and then diluted to 100 mL) solution was added to aliquot and then titrated against ferrous ammonium sulfate solution until the contents of blue-green color changed to reddish blue. Simultaneously, a blank (distilled water) was treated in a similar manner. The COD was expressed in mg L^−1^ and calculated using the formula,

$${\text{COD }}\left( {{\text{mg}}/{\text{l}}} \right) \, = \frac{{Vt_{\text{b}} - Vt_{\text{s}} \times N \times E \times 1000 }}{{V_{\text{s}} }},$$

where *E* = equivalent weight of oxygen, $V_{\text{s}}$ = volume of sample taken in mL, $Vt_{\text{b}}$ = blank titer value in mL, $Vt_{\text{s}}$ = titer value of sample in mL, *N* = normality of ferrous ammonium sulfate solution.

### Macronutrients estimation

The depletion of macronutrients, such as nitrogen (ammoniacal and nitrate) and phosphorus, in wastewater, was estimated using probe photometer kits and tablets (Palintest 7500 Photometer, USA) according to the supplier’s protocol described below.

### Ammoniacal nitrogen

The test tube was filled with samples up to the 10-mL mark. Then one ammonia No. 1 tablet and one ammonia No. 2 tablet were added, crushed and mixed to dissolve. For color development, the test tube was allowed to stand for 10 min and the reading was taken at 640 nm on a photometer. The obtained readings were multiplied by 1.3.

### Nitrate

The sample was filled up to 20 mL mark in the Nitratest tube. Then one level spoonful of Nitratest powder and one Nitratest tablet (do not crush) were added, afterward, the screw cap was tightened and shaken well for 1 min. The tube was allowed to stand for 1 min, then inverted gently for three or four times to aid flocculation. Afterward, the tube was allowed for 2 min to ensure settlement. From the 20-mL Nitratest tube, 10 mL of sample was filled up to the mark. Then, one Nitricol tablet was added, crushed, and mixed to dissolve. The test tube was allowed to stand for 10 min for color development and the reading was taken at 640 nm on a photometer. The obtained results were multiplied by 4.4.

### Phosphate

The test tube was filled with sample up to the 10 mL mark. Then one phosphate SR tablet and one phosphate HR tablet were added, crushed and mixed to dissolve one by one. For the color development, the test tube was allowed to stand for 10 min and the reading was taken at Photo 29 on a photometer. The obtained readings were multiplied by 1.3.

#### Cyanide

Cyanide content in raw and decontaminated SWW was determined using a modified picric acid method [53]. The wastewater sample was centrifuged at 15,000 rpm for 10 min (4 °C) and an aliquot of 50 µL was taken in a test tube. Then, 100 µL of picric acid (0.5% (w/v) picric acid) and 0.25 M Na_2_CO_3_ were added. The mixture was boiled for 5 min and then cooled down to room temperature (30 min). The final volume was made to 1 mL and absorbance was read at 520 nm using a spectrophotometer against a blank of distilled water and picric acid reagent. The cyanide removal efficiency was calculated as given below:

$${\text{Cyanide removal efficiency}}, \% = \frac{{{\text{Initial cyanide }}\left( {\text{mg/L}} \right) - {\text{Final cyanide }}\left( {\text{mg/L}} \right)}}{{{\text{Initial cyanide }}\left( {\text{mg/L}} \right) }} \times 100.$$

### Statistical analysis

All experiments were performed in triplicate and the average values with standard deviation were considered. Duncan’s multiple range test (DMRT) was conducted in Microsoft Excel 2010, and *p *<0.05 was considered significant.

## Data Availability

All data generated or analyzed during this study are included in this published article.

## Abbreviations

BLAST: Basic Local Alignment Search Tool
BOD: Biological oxygen demand
C:N: Carbon:nitrogen
COD: Chemical oxygen demand
EC: Electrical conductivity
FAME: Fatty acid methyl esters
FTIR: Fourier transform-infrared
GC-FID: Gas chromatography-flame ionization detector
GWP: Global warming potential
HCN: Hydrogen cyanide
MUFA: Monounsaturated fatty acids
NCBI: National Center for Biotechnology Information
NH_4_: Ammoniacal
NO_3_: Nitrate
PCR: Polymerase chain reaction
PO_4_: Phosphate
PUFA: Polyunsaturated fatty acids
SCO: Single-cell oil
SFA: Saturated fatty acid
SWW: Sago wastewater
TAG: Triacylglycerides
TDS: Total dissolved solids
TN: Total nitrogen
TS: Total solids
UASB: Upflow anaerobic sludge blanket
YEME: Yeast extract malt extract
